# Parentage testing and looking for single nucleotide markers associated with antler quality in deer (*Cervus elaphus*)

**DOI:** 10.5194/aab-65-267-2022

**Published:** 2022-07-28

**Authors:** Edith Elblinger, Julianna Bokor, Árpád Bokor, Vilmos Altbäcker, János Nagy, József Szabó, Bertalan Sárdi, Adrian Valentin Bâlteanu, Zsolt Rónai, László Rózsa, József Rátky, István Anton, Attila Zsolnai

**Affiliations:** 1 Kaposvár Campus, Hungarian University of Agriculture and Life Sciences, Kaposvár, 7400, Hungary; 2 Game Management Landscape Center, Hungarian University of Agriculture and Life Sciences, Kaposvár Campus, Bőszénfa, 7475, Hungary; 3 Institute of Life Sciences, University of Agricultural Sciences and Veterinary Medicine, Cluj-Napoca, Romania; 4 Department of Medical Chemistry, Molecular Biology and Pathobiochemistry, Eötvös Loránd University, Budapest, 1053, Hungary; 5 Kaposvár Campus, Hungarian University of Agriculture and Life Sciences, Herceghalom, 2053, Hungary; 6 Department of Obstetrics and Food Animal Medicine Clinic, University of Veterinary Medicine Budapest, Budapest, 1078, Hungary; 7 Institute for Farm Animal Gene Conservation, National Centre for Biodiversity and Gene Conservation, Gödöllő, 2100, Hungary

## Abstract

To provide a cost-efficient parentage testing kit for red deer (*Cervus elaphus*), a 63 SNP set has been developed from a high-density Illumina BovineHD BeadChip containing 777 962 SNPs after filtering of genotypes of 50 stags. The successful genotyping rate was 38.6 % on the chip. The ratio of polymorphic loci among effectively genotyped loci was 6.5 %. The selected 63 SNPs have been applied to 960 animals to perform parentage control. Thirty SNPs out of the 63 had worked on the OpenArray platform. Their combined value of the probability of identity and exclusion probability was $4.9\times 10^{-11}$ and 0.99803, respectively.

A search for loci linked with antler quality was also performed on the genotypes of the above-mentioned stags. Association studies revealed 14 SNPs associated with antler quality, where low-quality antlers with short and thin main beam antlers had values from 1 to 2, while high-quality antlers with long and strong main beams had values between 4 and 5. The chance for a stag to be correctly identified as having high-value antlers is expected to be over 88 %.

## 1 Introduction

Molecular genetic information was already used successfully in different species, e.g. for the determination of the origin of modern cattle (Beja-Pereira et al., 2006), for analysis of genetic diversity of horses (Aberle et al., 2004), and classification purposes in pigs (Zsolnai et al., 2006). Availability of SNP chip technology triggered its application in population genetics (Lancioni et al., 2016; Zsolnai et al., 2020a) and in association studies (Zsolnai et al., 2020b).

In deer, comprehensive studies have been conducted in search of quantitative trait loci (Slate et al., 2002) and for the determination of the inbreeding effect on breeding success (Slate et al., 2000). Molecular data have helped to confirm the genetic integrity of Carpathian red deer (Feulner et al., 2004) and were used to seek key genes in antler development (Molnár et al., 2007). The mechanism of antler development is also described by Gyurján et al. (2007) and Stéger et al. (2010).

One aspect of methodologies is the application of a paternity test, which is already routinely implemented for example in cattle and sheep (Glowatzki-Mullis et al., 1995, 2007) in many countries. There is an obvious demand from wildlife management and deer breeders to follow up the lineage, especially when important traits (antler characteristics, body weight, etc.) are logged in the herd book. The molecular genetic approach has already been implemented for completing this task previously. Bonnet et al. (2002) applied 11 microsatellites in three multiplex reactions, and Haanes et al. (2005) used 25 loci in six reactions for genotyping in deer.

In Hungary, two groups have developed multiplex microsatellite tests for parentage testing. Zsolnai et al. (2009) used nine loci in one reaction, and Szabolcsi et al. (2014) applied 10 loci in two reactions. Their obtained probability of identity was $1\times 10^{-11}$ and $1\times 10^{-12}$ , respectively. Since deer is an attractive game animal and research methodologies are becoming more advanced, studies – including fitness variation, performance prediction, or comparative population analysis – have been performed using Bovine BeadChip on deer (Kasarda et al., 2014). A total of 136 SNPs selected from the commercially unavailable Illumina 50K CervusSNP50 chip have already been developed for the New Zealand deer industry (Rowe et al., 2015). Such chip development or SNP discovery is on its way in Hungary and can be based on a genome map of red deer, CerEla1.0 (Bana et al., 2018).

Here, we aimed to select and test markers suitable for parentage testing in red deer based on Illumina Bovine HD SNP chip and OpenArray platforms to reduce the costs and labour of microsatellite genotyping. We also aimed to perform a genome-wide association study, to look for loci associated with antler quality, which can contribute to a more effective breeding strategy.

The selection of the Illumina Bovine SNP chip was based on its commercial availability and the high similarity of the bovine and deer genomes (Bana et al., 2018).

## 2 Materials and methods

We collected 49 red deer blood samples of stags from 10 places, including Baltacím (46 ${}^{\circ }$ 14 ${}^{\prime }$ 46 ${}^{\prime \prime }$ N, 17 ${}^{\circ }$ 49 ${}^{\prime }$ 32 ${}^{\prime \prime }$ E), Cserhát (46 ${}^{\circ }$ 14 ${}^{\prime }$ 00 ${}^{\prime \prime }$ N, 17 ${}^{\circ }$ 48 ${}^{\prime }$ 20 ${}^{\prime \prime }$ E), Égeres (46 ${}^{\circ }$ 14 ${}^{\prime }$ 34 ${}^{\prime \prime }$ N, 17 ${}^{\circ }$ 48 ${}^{\prime }$ 59 ${}^{\prime \prime }$ E), Homokos gödör (46 ${}^{\circ }$ 14 ${}^{\prime }$ 20 ${}^{\prime \prime }$ N, 17 ${}^{\circ }$ 49 ${}^{\prime }$ 06 ${}^{\prime \prime }$ E), Koronafürt felső (46 ${}^{\circ }$ 14 ${}^{\prime }$ 37 ${}^{\prime \prime }$ N, 17 ${}^{\circ }$ 49 ${}^{\prime }$ 47 ${}^{\prime \prime }$ E), Kuszó lucerna jobb alsó (46 ${}^{\circ }$ 14 ${}^{\prime }$ 11 ${}^{\prime \prime }$ N, 17 ${}^{\circ }$ 48 ${}^{\prime }$ 24 ${}^{\prime \prime }$ E), Kuszó lucerna jobb felső (46 ${}^{\circ }$ 14 ${}^{\prime }$ 21 ${}^{\prime \prime }$ N, 17 ${}^{\circ }$ 48 ${}^{\prime }$ 17 ${}^{\prime \prime }$ E), Szent-Tamás (46 ${}^{\circ }$ 14 ${}^{\prime }$ 19 ${}^{\prime \prime }$ N, 17 ${}^{\circ }$ 48 ${}^{\prime }$ 50 ${}^{\prime \prime }$ E), Templom Dél Kelet (46 ${}^{\circ }$ 14 ${}^{\prime }$ 17 ${}^{\prime \prime }$ N, 17 ${}^{\circ }$ 48 ${}^{\prime }$ 41 ${}^{\prime \prime }$ E), and Templom Dél Nyugat (46 ${}^{\circ }$ 14 ${}^{\prime }$ 14 ${}^{\prime \prime }$ N, 17 ${}^{\circ }$ 48 ${}^{\prime }$ 33 ${}^{\prime \prime }$ E) to perform SNP chip genotyping on the Illumina BovineHD BeadChip containing 777 962 SNPs. Sampling on different sites offers elimination of the formation of subgroups due to family structure, and it decreases the chance of population stratification. Selection criteria for stags were to represent high or low antler quality values within each sampled area to search for and elucidate the difference in antler quality. Sampling was performed by trained veterinarians during routine sample collection organized independently from this study. Sampling followed standard procedures and relevant national guidelines to ensure appropriate animal care.

Antler quality values were determined by a scoring method applied in the Bőszénfa deer farm, Hungary. Low-quality antlers (short and thin main beam antlers) reached a value from 1 to 2, while high-quality antlers (long and strong main beam) had values between 4 and 5. The low- and high-quality groups consisted of 24 and 25 animals, respectively. The preferred phenotype for the antler is to have long beams, high mass, and symmetry. Samples were prepared and genotyped by Neogen Europe Ltd. (Scotland, UK).

For parentage testing, the hair roots of 960 animals were collected. DNA from hair root samples was extracted by the Chelex (Bio-Rad, USA) procedure; 8–12 hair root samples were immersed into a 100 µ L, 5 % Chelex mixture, incubated overnight at 56 ${}^{\circ }$ C, and treated 10 min at 96 ${}^{\circ }$ C. The 63 SNPs, selected from BovineHD SNP chip, were genotyped on the OpenArray (Life Technologies, USA) platform. The surrounding sequences of the SNPs used for the design of primers and probes are available from https://webdata.illumina.com/downloads/productfiles/bovinehd/bovinehd-b1-annotation-file.zip (last access: 22 July 2022) and available in Supplement Table S1.

After filtering (part a) 777 962 loci – presented on the Illumina BovineHD BeadChip – for a call rate >0.95 , there were two alleles with MAF >0.05 . Additional filtering (part b) was included in the case of selection of SNPs suitable for parentage testing: Hardy–Weinberg equilibrium p value higher than 0.001, and MAF had to be over 0.3. For the association study, only part a of filtering was applied. The sequences of the loci found in GWAS are in Supplement Table S2.

Statistical analyses were performed by SVS SNP & Variation Suite 8.8.1. software (Golden Helix, Bozeman, MT, USA).

The performance of the SNPs selected for parentage testing was characterized by GenAlEx 6.5 (Peakall et al., 2012). For correction of population structure, the genomic kinship matrix was used in a multi-locus mixed model (Segura et al., 2012). The model was

1 y=Xβ+Zu+e,

where y is the antler quality score, X is the matrix of fixed effects composed of SNPs and covariates (date, place of birth, and the father), Z is the matrix of random animal effects, e means the residual effects, and β and u are vectors representing coefficients of fixed and random effects, respectively.

SVS and the PLINK software v.1.9 (Purcell et al., 2007) were used to build a multidimensional scaling (MDS) plot using a genome-wide identity-by-state pairwise distance matrix (mds-plot 2 and cluster options). Population stratification was calculated by SVS as described by Price et al. (2006). Python 3.6 programming language (Van Rossum and Drake, 2009) and the matplotlib 3.2.1 library (Hunter, 2007) were used for the visualization of PCA data.

Classification procedures were performed by Geneclass2 software (Piry et al., 2004).

## 3 Results

To achieve the goal to select SNPs suitable for pedigree control, we tested 777 962 SNPs on 50 red deer stags. After filtering for call rate and minor allele frequency, the number of retained loci was 103 562 and 25 919, respectively (filtering part a). After additional filtering (filtering part b) for LD pruning and Hardy–Weinberg equilibrium (7146 and 583 loci, respectively), the SNPs proved to be adequate for statistical analyses.

**Figure 1:**
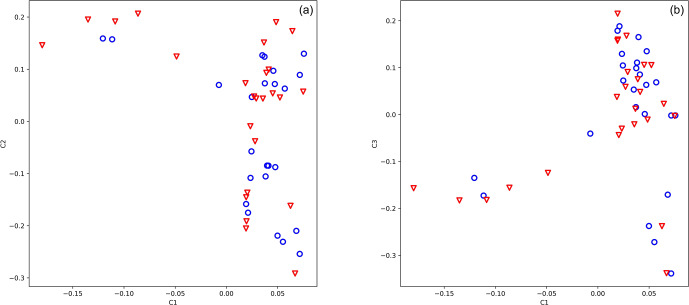
Principal component analysis of 49 stags based on their 583 loci. The blue circle shows animals with low antler quality, and the red triangle shows animals with high antler quality. Eigenvalues of axes C1 and C2 are 5.422 and 3.116, respectively.

The genotypic principal component analysis of both 25 919 and 583 SNPs did not reveal population stratification according to antler quality (Fig. 1a, b, 583 loci). Eigenvalues of axes 1, 2, and 3 were 5.422, 3.116, and 2.339, respectively.

We selected 63 SNPs for parentage testing. The probability level for the identity of this set was $7\times 10^{-24}$ . The surrounding sequence of selected SNPs was used to design primers and probes by OpenArray Product Configurator (Life Technologies, USA) for genotyping 960 samples on the OpenArray platform (Life Technologies, USA). The callable 30 SNPs (Supplement Table S1) out of the selected 63 SNPs reached the $4.9\times 10^{-11}$ value of the probability of identity on 960 animals.

**Figure 2:**
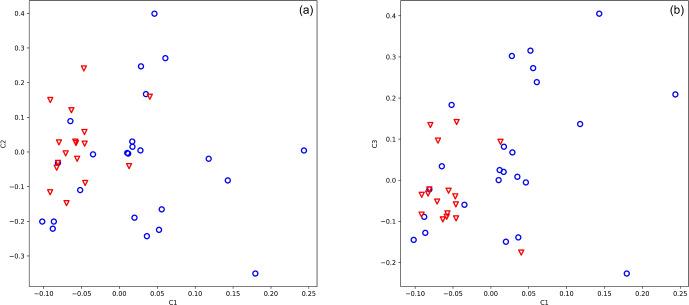
Principal component analysis of 49 stags based on their 14 loci selected to differentiate by antler quality. The blue circle shows animals with low antler quality, and the red triangle shows animals with high antler quality. Eigenvalues of axes C1 and C2 are 9.857 and 5.637, respectively.

In GWAS analysis date and place of birth and the father of the animal were included in the model. A genomic kinship matrix was used to correct possible relatedness, and 14 SNPs (Supplement Table S2) out of 25 919 displayed significant differentiation between the two groups (high and low antler quality). Eigenvalues of PCA coordinates were 9.857, 5.637, and 5.004 for axes 1, 2, and 3. The selected SNPs were able to position high- and low-antler-value animals into two distinct groups (Fig. 2a, b).

Depending on the algorithm chosen within GeneClass2 software (Piry et al., 2004) the number of misclassified animals (from the low-value group) ranged from 3 to 6, which is 6 %–12 % of the total number of individuals.

## 4 Discussion

Haynes and Latch (2012) have achieved a 38.7 % successful genotyping rate on *Odocoileus hemionus* and *Odocoileus virginianus* using Illumina Bovine BeadChip and they found 5 % polymorphic loci among the successfully genotyped SNPs on the Bovine BeadChip. In our case on *Cervus elaphus*, 13.3 % of the genotyped loci were effective, while the ratio of polymorphic loci among effectively genotyped loci was 6.5 %. The selected 63 SNPs for parentage testing had an identity value ( $7\times 10^{-24})$ that was similar to the value of SNP sets used in white pigs (Rohrer et al., 2007) or in Mangalitza pigs (Zsolnai et al., 2013) ( $1\times 10^{-23})$ . Genotyping 960 animals on the OpenArray platform, only 30 SNPs out of 63 were callable. The $4.9\times 10^{-11}$ value of the probability of identity of these 30 SNPs is similar to the previous $1\times 10^{-11}$ (Zsolnai et al., 2009) or $1\times 10^{-12}$ values (Szabolcsi et al., 2014) of deer microsatellite sets. The exclusion probability of the reported 30 SNPs is 0.99803. As for microsatellite sets, this value is slightly better than the previously reported 0.99630 (Zsolnai et al., 2009) and lower than 0.99999 (Szabolcsi et al., 2014). The price of typing 63 SNPs was about a third of the price of microsatellite genotyping and required less working time. By switching from OpenArray (Life Technologies, USA) to another genotyping platform, the number of callable SNPs and the exclusion probability value could be increased to 0.99999.

For the genome-wide association study, we used data acquired from 50 animals where antler quality was known. The genotypic principal component analysis of 583 SNPs did not divide the population by antler quality (Fig. 1a, b). The lambda value calculated by SVS was 1.02, indicating that there was no hidden population stratification among the sampled animals.

After filtering (part a) we found 14 SNPs that are associated with antler values (Supplement Table S2). Previously such a GWA-aided search was successfully applied in the case of Mangalitza pigs to find trait-associated loci for checking quality and quantity parameters in the meat industry (Szántó-Egész et al., 2013, 2016).

Evaluating our 14 SNPs on red deer by PCA (Fig. 2) and classification algorithms implemented in GeneClass2 software (Piry et al., 2004), we have found that animals with a high antler value have never fallen into the low-value group. However, several animals (4 %–10 %) from the low-value group have been assigned to the high-value group.

Several candidate genes have been found in the close vicinity of the antler-quality-associated markers (Supplement Table S2). Among the candidate genes, we are mentioning those where bone- or antler-related functions are known. Sentrin-specific protease 1 (*SENP1*) markedly enhances androgen-receptor-mediated transcription in males, mediates cell growth and differentiation, and maintains male reproductive functions (Cheng et al., 2004). Collagen alpha-1(II) chain (*COL2A1*) has key roles in chondrogenesis and osteogenesis (Jia et al., 2021); it is upregulated in the main beams of antlers (Yao et al., 2020). Vitamin D receptor (*VDR*) alleles are associated with postmenopausal osteoporosis susceptibility and bone mineral density (Zhang et al., 2018). Dynamin 1 like (*DNM1L*) belongs to a superfamily of GTPases, which are related to osteoclast differentiation and bone loss (Jeong et al., 2021). Tyrosyl-TRNA synthetase 2 (*YARS2*) is coupled with the impaired ability of the bone marrow to produce normal red blood cells (Riley et al., 2013). Plakophilin 2 (*PKP2*) overexpression was able to stop the proliferation of osteosarcoma (He et al., 2021). Zinc finger protein 518B (*ZNF518B*) is a putative ageing modulator (Sleiman et al., 2020). WD repeat-containing protein 1 (*WDR1*) is part of a deer antler extract (Yao et al., 2019). Solute carrier family 2 member 9 (*SLC2A9*) plays a role in gout, a form of rheumatic arthritis (Merriman and Dalbeth, 2011), and has a significant role in glucose homeostasis. Among the enriched targets of miRNAs there is zinc finger and BTB domain containing 49 (*ZBTB49*) after a growth factor-beta 1 stimulation (Ong et al., 2017). The latter compound has a high impact on bone formation and resorption (Bonewald et al., 1990). Molybdenum cofactor sulfurase (MOCOS) is involved in purine metabolism (Kurzawski et al., 2012), and extracellular purine is vital for bone homeostasis (Agrawal and Jørgensen, 2021). Elongation protein 2 (*ELP2*) has a role in osteogenesis and osteogenic differentiation (Wu et al., 2021). Exocyst complex component 6B (*EXOC6B*) is associated with joint dislocation syndrome (Girisha et al., 2016). Nucleoporin 58 (*NUP58*) mediates molecular trafficking to and from the nucleus and can be tied to delayed abscission during mitosis (Hartono et al., 2019). Spermatogenesis associated 13 (*SPATA13*) can weaken the migration of type I collagen via activation of GTPase (Jean et al., 2013). Zinc finger homeobox 4 (*ZFHX4*) orchestrates endochondral bone formation (Nakamura et al., 2021). Lysine demethylase 4C (*KDM4C*) regulates condensin-dependent heterochromatin reorganization and is connected with deterioration and premature bone ageing (Huang et al., 2019). Glycine decarboxylase (*GLDC*) locus is associated with overall survival in patients with osteosarcoma (Lin et al., 2020). Glutathione peroxidase (GPX) level is higher at the upper sections of the velvet antler and associated with lower Ca content (Cheng et al., 2017). SMAD family member 7 (*SMAD7*) directly interacts with microtubule actin crosslinking factor 1, initiating downstream osteogenic pathways (Zhao et al., 2020). Dymeclin (DYM) is an important part of the extracellular matrix and has a role in bone development (Denais et al., 2011). Among the differentially abundant metabolites of ageing bone marrow mesenchymal stem cells, acetyl-CoA acyltransferase 2 (*ACAA2*) is related to senescence and lipid metabolism (Yu et al., 2022).

The approach for finding selection markers for antler quality can be enhanced by (i) incorporating more animals into the study, (ii) development of a deer SNP chip based on red deer populations existing in Hungary and on existing whole-genome sequencing (Bana et al., 2018), and (iii) collecting blood at different stages of antler development and merging genotypic, phenotypic, and blood-metabolite data for deeper analysis.

As for parentage testing, the identified 30 SNPs produce similar discriminating power to that of a microsatellite set (Zsolnai et al., 2009). The advantage of SNP typing lays in its cost-effectiveness compared to microsatellites.

As for antler quality predictions, the listed 14 SNPs coupled with antler quality can be used to identify those animals at birth that are supposed to develop good-quality antlers in their life. The chance that an animal (preselected by SNP typing) has a desirable high-valued antler is expected to be over 88 %.

## Supplement

10.5194/aab-65-267-2022-supplement The supplement related to this article is available online at: https://doi.org/10.5194/aab-65-267-2022-supplement.

## Data Availability

The surrounding sequences of the SNPs described in the MS are available in the Supplement.
