# Supplementary material for: Parentage testing and looking for single nucleotide markers associated with antler quality in deer (Cervus elaphus)
Source: Arch Anim Breed. 2022 Jul 28;65(3):267–74. doi: 10.5194/aab-65-267-2022 (PMC9399935; doi:10.5194/aab-65-267-2022)
Supplement: The supplement related to this article is available online at: https://doi.org/10.5194/aab-65-267-2022-supplement. [file aab-65-267-supplement.zip › aab-65-267-2022-supplement-title-page.pdf]

Supplement of Arch. Anim. Breed., 65, 267–274, 2022
https://doi.org/10.5194/aab-65-267-2022-supplement
© Author(s) 2022. CC BY 4.0 License.

Open Access
Archives Animal Breeding

Supplement of

Parentage testing and looking for single nucleotide markers associated with antler quality in deer (*Cervus elaphus*)

Edith Elblinger et al.

Correspondence to: Attila Zsolnai (attila.zsolnai@gmail.com)

- aab-65-267-2022-supplement-title-page.pdf
- SupplementaryTables_sequences_genes.xlsx

The copyright of individual parts of the supplement might differ from the article licence.
